# Endotheliopathy and Platelet Dysfunction as Hallmarks of Fatal Lassa Fever

**DOI:** 10.3201/eid2611.191694

**Published:** 2020-11

**Authors:** Lucy E. Horton, Robert W. Cross, Jessica N. Hartnett, Emily J. Engel, Saori Sakabe, Augustine Goba, Mambu Momoh, John Demby Sandi, Thomas W. Geisbert, Robert F. Garry, John S. Schieffelin, Donald S. Grant, Brian M. Sullivan

**Affiliations:** The Scripps Research Institute, La Jolla, California, USA (L.E. Horton, S. Sakabe, B.M. Sullivan);; University of Texas Medical Brach, Galveston, Texas, USA (R.W. Cross, T.W. Geisbert);; Tulane University School of Medicine, New Orleans, Louisiana, USA (J.N. Hartnett, E.J. Engel, R.F. Garry, J.S. Schieffelin);; Kenema Government Hospital, Kenema, Sierra Leone (A. Goba, M. Momoh, J.D. Sandi, D.S. Grant);; Ministry of Health and Sanitation, Freetown, Sierra Leone (A. Goba, M. Momoh, J.D. Sandi);; Eastern Polytechnic Institute, Kenema (M. Momoh, D.S. Grant);; Njala University, Moyamba, Sierra Leone (J.D. Sandi); University of Sierra Leone, Freetown (D.S. Grant)

**Keywords:** Lassa fever, hemostasis, platelet, protein C, coagulation, fibrinolysis, viruses, vector-borne infections, zoonoses, hemorrhagic fever

## Abstract

Lassa fever (LF) causes multisystem disease and has a fatality rate <70%. Severe cases exhibit abnormal coagulation, endothelial barrier disruption, and dysfunctional platelet aggregation but the underlying mechanisms remain poorly understood. In Sierra Leone during 2015–2018, we assessed LF patients’ day-of-admission plasma samples for levels of proteins necessary for coagulation, fibrinolysis, and platelet function. P-selectin, soluble endothelial protein C receptor, soluble thrombomodulin, plasminogen activator inhibitor 1, ADAMTS-13, von Willebrand factor, tissue factor, soluble intercellular adhesion molecule 1, and vascular cell adhesion molecule 1 were more elevated in LF patients than in controls. Endothelial protein C receptor, thrombomodulin, intercellular adhesion molecule 1, plasminogen activator inhibitor 1, D-dimer, and hepatocyte growth factor were higher in fatal than nonfatal LF cases. Platelet disaggregation occurred only in samples from fatal LF cases. The impaired homeostasis and platelet dysfunction implicate alterations in the protein C pathway, which might contribute to the loss of endothelial barrier function in fatal infections.

Lassa fever (LF) is an acute viral hemorrhagic fever endemic to West Africa, where »300,000–500,000 cases/year occur and mortality rates are high (*1*). Humans primarily are infected from exposure to excreta from the rodent host, Natal multimammate mouse (*Mastomys natalensis*). Pregnant women especially are susceptible to severe disease; infection during pregnancy usually leads to spontaneous abortion (*2*). The high rates illness and death, lack of vaccines or approved treatments, and potential to cause a public health emergency led to the US Centers for Disease Control and Prevention to classify LF as a category A bioterrorism agent (https://emergency.cdc.gov/agent/agentlist-category.asp) and the World Health Organization to classify LF as a priority disease (https://www.who.int/dg/priorities).

Early clinical manifestations and symptoms of LF often are nonspecific and easily confused with other diseases prevalent in the endemic region, such as malaria and typhoid fever. Consequently, patients often are not admitted for LF treatment until symptoms are severe and they have failed other therapies. Bleeding at mucous membranes and edema generally are seen in the most severe cases (*3*,*4*), but bleeding diathesis reportedly was common during a recent outbreak in Nigeria (*4*). Overt hemorrhage is rare, mostly limited to the mucosal surfaces, and not severe enough to cause shock. Pathologic changes seen on autopsy lack major cell and tissue injury but include signs of pleural effusion, pulmonary edema, ascites, and gastrointestinal mucosa bleeding (*5*,*6*), all indications of systemic vascular leakage. Severely ill LF patients often have mild to moderate thrombocytopenia, but rarely have platelet counts <100,000/μL (*7*). Thrombocytopenia is a common feature of hemorrhagic fevers and vascular permeability disorders (*8*), but the decrease in platelet counts in acute LF is not low enough to cause spontaneous hemorrhage.

LF could be characterized as a disease of enhanced vascular permeability but the underlying pathophysiology remains ambiguous. Because gross signs of endotheliopathy and vascular leakage are restricted mostly to severe LF cases, we hypothesized that differences in hemostatic markers between fatal and nonfatal cases found in day-of-admission plasma samples could be prognostic and elucidate changes in hemostasis during LF. We identified differences in markers of endothelial activation and injury between fatal and nonfatal cases that indicate disruption of the protein C pathway and endothelial stress in fatal LF.

## Materials and Methods

### Subjects

The study was conducted at the Lassa Laboratory at the Kenema Government Hospital (KGH) in Sierra Leone during 2015–2018. Patients with diagnosed acute LF met clinical criteria (*9*). We confirmed LF by detecting Lassa fever virus (LASV) antigen (LASV-Ag) by using ReLASV Pan-Lassa Antigen (Zalgen Labs, https://www.zalgen.com) or by detecting Lassa-specific IgM (LASV IgM) by using ReLASV Pan-Lassa IgG/IgM (Zalgen Labs) ELISA tests. All human subjects provided written informed consent before inclusion in the study. The study was approved by the institutional review boards of the Scripps Research Institute (approval no. 17-6972), Tulane University (approval no. 09-00419), and the Sierra Leone Ethics and Scientific Review Committee.

### Clinical Information

Blood was collected in EDTA tubes, processed within 6 hours, and stored at −20°C until analysis. Clinical data, including blood chemistries, liver function tests, and blood counts were obtained when feasible by using Piccolo Xpress (Abaxis, https://www.abaxis.com) and manual complete blood counts. Clinical outcomes data was incomplete and survival status was not known for patients not admitted to the viral hemorrhagic fever ward or transferred to another medical facility.

### Biosafety

Samples were brought to the Lassa Laboratory from the Lassa ward in secondary containers. Staff performing experiments in the laboratory wore full personal protective equipment, including Tyvec suits, N95 masks, face shields, gloves, and boots. Infectious samples were handled in Biosafety cabinets.

### ELISAs

We assessed plasma samples by using commercially available kits. For plasminogen activator inhibitor 1 (PAI-1) we used Human Serpin E1/PAI-1 Quantikine ELISA Kit (R&D Systems, https://www.rndsystems.com) or Human PAI-1 Platinum Kit (eBioscience, https://www.thermofisher.com). We used Human t-Plasminogen Activator/tPA Quantikine ELISA Kit (R&D Systems) to measure tissue plasminogen activator (tPA). We used Human Thrombomodulin/BDCA-3 Quantikine ELISA Kit (R&D Systems) or Human Thrombomodulin ELISA Kit (Innovative Research Inc., https://www.innov-research.com) to measure thrombomodulin (THBD) and Human Thrombin-Antithrombin Complex ELISA Kit (Abcam, https://www.abcam.com) to measure thrombinantithrombin (TAT) complexes. To assess endothelial protein C receptor (EPCR) we used Human EPCR DuoSet ELISA Kit (R&D Systems), for D-dimer we used Human D-Dimer ELISA Kit (Abcam), for a disintegrin and metalloproteinase with a thrombospondin type 1 motif, member 13 (ADAMTS-13) we used Human ADAMTS13 Quantikine ELISA Kit (R&D Systems), for P-selectin we used Human CD62P ELISA Kit (Abcam), for hepatocyte growth factor (HGF) we used Human HGF Quantikine ELISA Kit (R&D Systems), for von Willebrand factor (vWF) we used vWF Human ELISA Kit (ThermoFisher, https://www.thermofisher.com), and for tissue factor we used Human Coagulation Factor III/Tissue Factor Quantikine ELISA Kit (R&D Systems).

### Platelet Aggregation Studies

Cryopreserved plasma samples were dialyzed in phosphate-buffered saline by using a 100 kDa or 1 MDa pore membrane (Spectra-Por Float-A-Lyzer; Sigma-Aldrich, https://www.sigmaaldrich.com) to remove EDTA and mixed 1:1 with platelet rich plasma collected from a healthy subject. Light transmission aggregation was performed on a Chrono-Log 450 (Chrono-Log, https://www.chronolog.com) aggregometer by using AGGRO/LINK 8 software (Chrono-Log). To initiate platelet aggregation, we used 5 μM adenosine diphosphate and incubated for 1 min.

### Statistical Analyses

**Figure 1:**
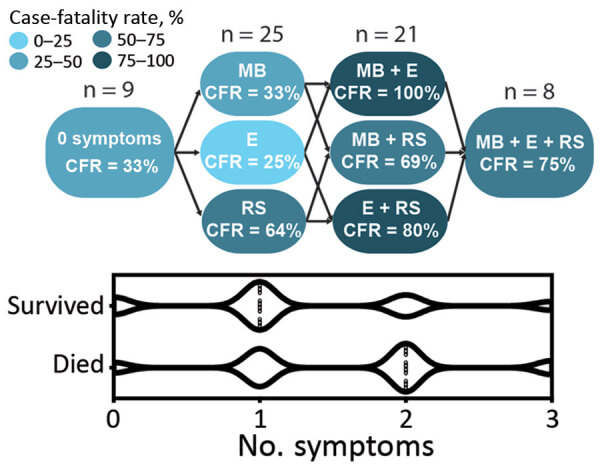
Clinical features suggestive of vascular permeability in patients with Lassa fever, Sierra Leone, 2015–2018. Patients with acute LF who had >2 signs or symptoms indicating vascular permeability at the time of admission were more likely to have fatal outcomes based on multiple logistic regression compared with patients who had no of symptoms vascular permeability (p = 0.0335). Case-fatality rates associated with various signs and symptoms are shown at the top and violin plot depicting the number of persons in each category at the bottom (median value indicated by dotted vertical lines). CFR, case-fatality rate; E, generalized edema; MB, mucosal membrane bleeding; RS, respiratory symptoms, such as cough or pulmonary edema.

We performed statistical analyses by using Prism (GraphPad Software, https://www.graphpad.com) and Excel (Microsoft Corporation, https://www.microsoft.com) software. We used a 1-way analysis of variance Kruskal-Wallis test for comparing >3 variables to determine whether group values were due to random sampling. Then, we subjected these data to Dunn’s multiple comparisons test to detect statistically significant differences between groups. We used Mann-Whitney tests when we assessed only 2 variables. We used linear regressions to find the best fit curve and Spearman correlations to find correlations between 2 variables. We used multiple logistical regression for some data (Figure 1) and considered p<0.05 statistically significant.

## Results

The biorepository at the KGH contains samples from consented patients admitted and not admitted to the Lassa ward, but clinical information and outcomes were not available for all samples used. LF was diagnosed in patients with symptoms plus a positive ELISA result indicating either LASV-Ag or LASV-specific IgM, as previously described (*9*). We collected clinical data and plasma samples from 98 LF patients, 33 non–LF febrile controls (NLFCs), and 13 healthy controls (HCs) and summarized demographic information, outcomes, and LASV-Ag or IgM status for patients whose data were available (Table).

**Table:** Characteristics and clinical and laboratory findings of patients in a study of Lassa fever fatality, Sierra Leone during 2015–2018*

Characteristics	All Lassa fever cases, n = 98	Outcomes		Non–LF febrile controls, n = 7
		Survived, n = 23	Died, n = 51	
Mean age, y (range)	23 (1–75)	20 (3–29)	25 (1–75)	24 (3–60)
Sex				
F	64 (65.3)	18 (78.3)	29 (56.8)	2 (22.2)
M	34 (34.7)	5 (21.7)	22 (43.1)	5 (71.4)
Clinical findings	n = 70	n = 23	n = 34	n = 9
Bleeding symptoms				
Bleeding gums	5 (7.1)	2 (8.7)	2 (5.9)	1
Epistaxis	4 (5.7)	1 (4.3)	2 (5.9)	1
Blood in stool	6 (8.6)	3 (13.0)	3 (8.8)	0
Blood in vomit	6 (8.6)	2 (8.7)	4 (11.8)	2
Injected conjunctiva	11 (15.7)	2 (8.7)	7 (20.6)	0
Bleeding hematoma	1 (1.4)	0	1 (2.9)	0
Blood in sputum	6 (8.6)	1 (4.3)	4 (11.8)	0
Blood in urine	3 (4.3)	2 (8.7)	1 (2.9)	1
Vaginal bleeding	3 (4.3)	1 (4.3)	2 (5.9)	1
Other bleeding	6 (8.6)	2 (8.7)	2 (5.9)	0
Edema	21 (30.0)	6 (26)	12 (35.3)	0
Other clinical symptoms				
Cough	45 (64.3)	15 (65.2)	24 (70.6)	6 (66.7)
Fever	54 (77.1)	16 (69.6)	30 (88.2)	0
Rash	2 (2.9)	0	2 (5.9)	1 (11.1)
Headache	60 (85.7)	20 (87)	28 (82.4)	6 (66.7)
Vomiting	37 (52.9)	11 (47.8)	20 (58.8)	5 (55.6)
Diarrhea	32 (45.7)	8 (34.8)	24 (70.6)	1 (11.1)
Jaundice	6 (8.6)	0	0	1 (11.1)
Laboratory findings, mean (+SD)				
AST, U/L	439 (+689)	423 (+651.7)	717 (+777)	76 (+78)
ALT, U/L	455 (+556)	246 (+463)	707 (+540)	41 (+35)
Alkaline phosphatase, U/L	220 (+269)	106 (+87)	313 (+339)	157 (+108)
Total bilirubin, mg/dL	1.18 (+1.38)	0.95 (+1.41)	1.44 (+1.54)	4.12 (+0.59)
Total protein, g/L	6.7 (+1.8)	7.1 (+10.0)	6.2 (+1.7)	7.3 (+0.5)
Creatinine, mg/dL	1.85 (+2.12)	0.88 (+0.40)	2.75 (+2.70)	0.5 (+0.26)
BUN, mg/dL	24 (+32)	10 (+5)	31 (+27)	17 (+11)
*Values are no. (%) except as indicated. ALT, alanine aminotransferase; AST, aspartate aminotransferase; BUN, blood urea nitrogen; LF, Lassa fever.				

Severe LF is characterized by facial and pulmonary edema, pleural effusions, and ascites, indicating profound vascular dysfunction. To a lesser extent, patients also might exhibit petechiae and mucosal membrane bleeding, which suggest dysregulation of the coagulation or fibrinolytic systems (*7*). Patients who died during our study more frequently had edema, bleeding at mucous membranes, and respiratory signs, including cough or hemoptysis, than patients who survived (Table). Patients who had >2 symptoms suggestive of vascular permeability had higher case-fatality rates than patients with <2 symptoms (Figure 1).

### Coagulation Markers 

**Figure 2:**
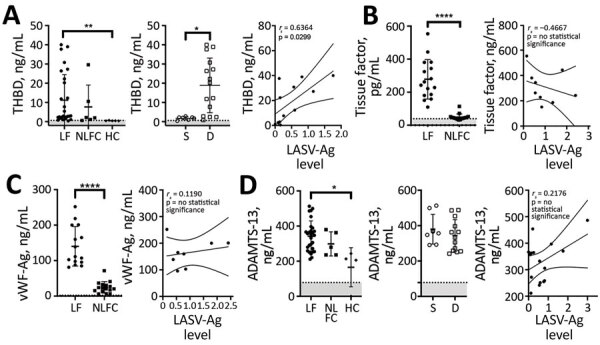
Laboratory findings for coagulation markers for patients with acute LF, NLFCs, and HCs, Sierra Leone, 2015–2018. A) Soluble thrombomodulin is elevated in LF and predicts fatal outcomes (Kruskal-Wallis p = 0.0002 across all groups). Levels of soluble THBD were statistically significantly higher (**p = 0.0084) in acute LF cases (n = 28) than in HCs (n = 5); patients who died (n = 15) had higher levels of soluble THBD than those who survived (n = 7; *p = 0.0239); and we noted a positive correlation between soluble THBD and LASV-Ag levels (n = 12). B) Tissue factor was statistically significantly elevated (****p<0.0001) in acute fatal LF cases (n = 16) compared with NLFC (n = 16), but no statistically significant correlation was found between TF and LASV-Ag levels in LF patients. C) vWF Ag levels were statistically significantly elevated (****p<0.0001) in acute fatal LF patients (n = 15) compared with NLFC (n = 16), but no statistically significant correlation was found between vWF and LASV-Ag levels in LF patients. D) Plasma levels of ADAMTS-13 were statistically significantly different between groups (Kruskal-Wallis p = 0.0155). Levels of ADAMTS-13 were statistically significantly higher (*p = 0.0292) in patients with acute LF (n = 28) compared with HCs (n = 4). No differences were seen between those who died (n = 13) versus those who survived (n = 8), nor was a statistically significant correlation found between ADAMTS-13 and LASV-Ag in LF patients. Limits of detection are indicated by dashed lines and gray shading below. Error bars show SDs; horizontal lines indicate means. D, died; HC, healthy control; LF, Lassa fever; LASV-Ag, Lassa fever virus antigen; NLFC, non-LF febrile control; S, survived; THBD, thrombomodulin; vWF, von Willebrand factor; vWF-Ag, von Willebrand factor antigen.

To characterize hemostatic changes in LF patients, we measured levels of proteins essential to hemostasis and compared these results with plasma from NLFCs and HCs. The procoagulant enzyme, thrombin, contributes to the formation of hemostatic clots by converting fibrinogen into fibrin, but thrombin also induces endothelial permeability (*10*). THBD, a cofactor located on the surface of endothelial cells, binds thrombin and changes its specificity from a procoagulant enzyme to an anticoagulant enzyme. THBD-bound thrombin activates protein C into activated protein C (APC). APC then inhibits further thrombin formation by inactivating factors Va and VIIIa. When the THBD ectodomain is cleaved from the transmembrane stack, soluble THBD (sTHBD) can be detected in plasma and is associated with endothelial cell activation and vascular dysfunction (*11*). We noted much higher levels of sTHBD (mean 11.18 ng/mL) in LF patients than in HCs (mean 0.48 ng/mL; p = 0.0084), consistent with generation of endothelial stress factors in LF-infected patients (Figure 2, panel A). Samples from fatal LF cases also had more elevated sTHBD levels (mean 18.94 ng/mL) than samples from LF survivors (mean 1.78 ng/mL; p = 0.0239), indicating that advanced vascular dysfunction is associated with fatality (Figure 2, panel A). We also found a positive correlation between LASV-Ag levels and sTHBD in LF patients (Spearman ρ [*r_s_*] = 0.6364; p = 0.0299).

Next, we measured levels of tissue factor (TF), the key initiator of coagulation in severe systemic infections that lead to sepsis (*12*). TF, a transmembrane glycoprotein, triggers the extrinsic coagulation cascade by binding factors VIIa and X and facilitating thrombin generation when exposed on the surface of the vascular endothelium at a site of injury (*13*). Circulating TF in microparticles can be released from leukocytes, endothelial cells, and platelets (*14*). Because hyperactivation of the TF system has been observed in sepsis (*15*) and disseminated intravascular coagulation (DIC), we measured soluble TF in plasma and found much higher levels (mean 278 pg/mL) among fatal LF cases than NLFCs (mean 48.8 pg/mL; p<0.0001) (Figure 2, panel B), but we did not find a correlation between TF and antigen levels among LF patients.

The endothelium constitutively releases low molecular weight vWF multimers. High molecular weight vWF multimers are released by activated platelets and endothelium and are highly biologically active and necessary for platelet adhesion, aggregation, and initiation of fibrin clot formation. We did not have specific equipment needed to measure high molecular weight vWF multimers, but we measured total vWF antigen (vWF-Ag) by ELISA and found vWF-Ag levels were statistically significantly higher (mean 140.2 ng/mL) in fatal LF cases than in NLFCs (mean 24.58 ng/mL; p<0.0001). vWF levels in LF patients did not correlate with LASV-Ag levels (Figure 2, panel C). 

We also measured levels of ADAMTS-13, the metalloprotease that degrades high molecular weight vWF multimers. Degradation of vWF can lead to increased bleeding because high molecular weight vWF multimers are critical for homeostasis (*16*). We found increased ADAMTS-13 levels (mean 342.4 ng/mL) in LF patients compared with HCs (mean 165.5 ng/mL; p<0.0292) (Figure 2, panel D), suggesting high molecular weight vWF multimers also might be decreased, especially in the microvasculature where shear stress enhances the availability of vWF cleavage sites. However, we did not observe differences in ADAMTS-13 levels between LF patients who survived (mean 379.8 ng/mL) and those who died (mean 343.6 ng/mL). We also found no statistically significant correlation between LASV-Ag levels and ADAMTS-13 (Figure 2, panel D).

### Fibrinolysis Markers 

**Figure 3:**
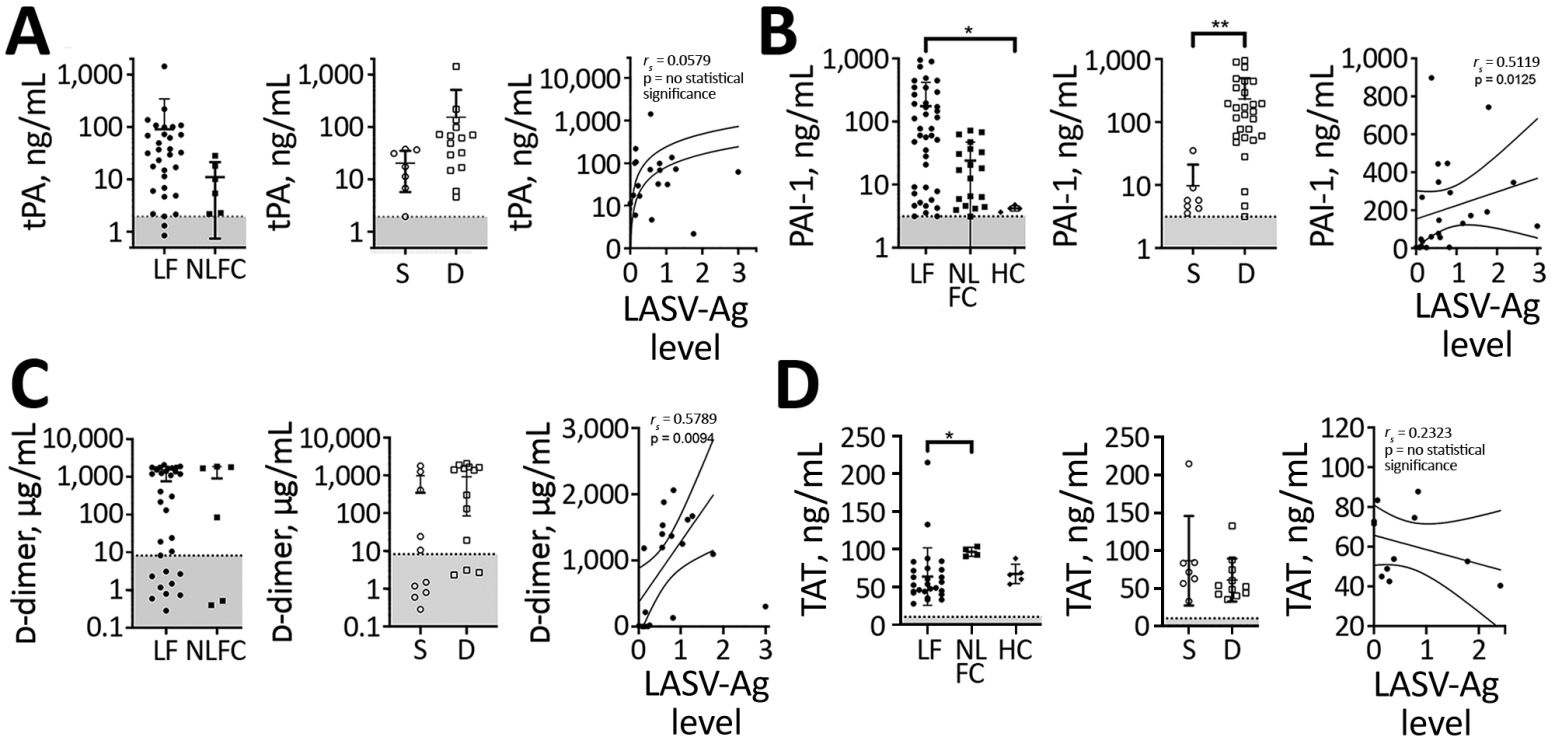
Laboratory findings for fibrinolysis markers for patients with acute LF, NLFCs, and healthy controls HCs, Sierra Leone, 2015–2018. A) Levels of tPA were not statistically significantly different between groups (Kruskal-Wallis p = 0.0516); no differences in tPA were observed between LF (n = 31) and NLFC (n = 6), or between patients who survived (n = 7) and patients who died (n = 15). B) PAI-1 levels predict fatal outcomes (Kruskal-Wallis p<0.0001 across all groups); PAI-1 was statistically significantly elevated (*p = 0.0145) in patients with acute LF (n = 40) compared with HCs (n = 5); patients who died (n = 28) had higher levels of PAI-1 (**p = 0.0031) than those who survived (n = 7); statistically significant positive correlation was observed between PAI-1 and LASV antigen in LF patients (n = 23). C) D-dimer levels in plasma were not statistically significantly different between groups (Kruskal-Wallis p = 0.2167); no difference in D-dimer levels were observed between LF (n = 32) and NLFC (n = 6), nor between patients who died (n = 13) and those who survived (n = 10); and a statistically significant positive correlation was observed between D-dimer levels and LASV antigen in LF patients (n = 19). D) No increased levels of TAT were observed between LF (n = 26) and HC (n = 5) (*p value), nor between patients who survived (n = 7) and died (n = 11), and no statistically significant correlation was observed between TAT levels and LASV-Ag in LF patients (n = 11). Limits of detection are indicated by dashed lines and gray shading below. Error bars show SDs; horizontal lines indicate means. D, died; HC, healthy control; LF, Lassa fever; LASV-Ag, Lassa fever virus antigen; NLFC, non-LF febrile control; PAI-1, plasminogen activator inhibitor 1; S, survived; TAT, thrombinantithrombin complexes; THBD, thrombomodulin; tPA, tissue plasminogen activator.

We also sought to determine changes in the fibrinolysis cascade. tPA, an enzyme that activates the zymogen plasminogen into the enzyme plasmin, dissolves fibrin clots and is inhibited by PAI-1. tPA levels trended higher (mean 90.7 pg/mL) in LF patients than NLFCs (median 11.04 pg/mL) but did not reach statistical significance (Figure 3, panel A). PAI-1 levels were much higher (mean 175.8 ng/mL) in LF patients than in HCs (mean 4.20 ng/mL; p = 0.0145). We also found a statistically significant positive correlation between LASV-Ag and PAI-1 (*r_s_* = 0.5119; p = 0.0125) (Figure 3, panel B). tPA has a short half-life in circulation; elevated tPA levels might not represent active tPA but tPA–PAI-1 complexes, which are not distinguished by our ELISA. Only PAI-1 was statistically significantly higher in patients with fatal LF (p = 0.0031) (Figure 3, panel B).

Increased fibrinolysis leads to the production of fibrin degradation products, such as D-dimers. We noted elevated D-dimers in »50% of LF patients and NLFCs, but we found no statistically significant differences between the groups. However, among LF patients, we found a statistically significant positive correlation between LASV-Ag levels and D-dimers (*r_s_* = 0.5789; p = 0.0094) (Figure 3, panel C). Increased D-dimer is one parameter used to determine DIC, which has been controversial in LF. Diagnosing DIC can be complex and requires measuring several parameters, including platelet count, prothrombin time, fibrin degradation products, and fibrinogen level, but we could not measure these with the available samples. Elevated TAT complexes are a common feature of DIC but do not indicate DIC when measured alone. TAT levels in LF patients were not elevated (mean 63.83 ng/mL) compared with HCs (mean 67.35 ng/mL) but were statistically significantly lower than levels in NLFCs (mean 96.64 ng/mL; p = 0.0349). We did not detect differences between TAT in LF survivors (median 71.47 ng/mL) and LF deaths (median 52.59 ng/mL), nor did we find a correlation between LASV-Ag antigen levels and TAT (Figure 2, panel D). We observed high baseline TAT values in HCs, but the levels were not different from baseline values reported in other studies that used the same ELISA kit (*17*,*18*), indicating differences in standard values could be assay specific.

**Figure 4:**
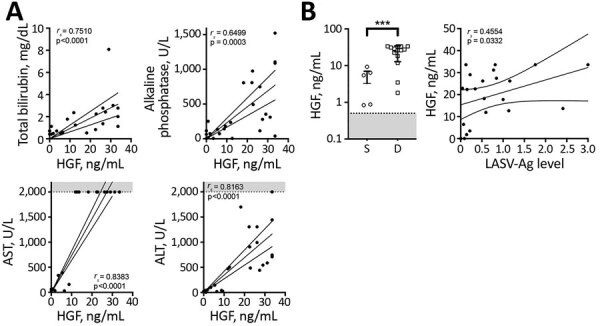
Hepatocyte growth factor plasma levels in patients with acute LF and liver function tests in acute LF cases, Sierra Leone, 2015–2018. A) HGF was positively correlated with total bilirubin (n = 23), alkaline phosphatase (n = 26), AST (n = 26), and ALT (n = 27). B) HGF levels in acute LF patients were statistically significantly higher (***p = 0.0005) in patients who died (n = 14) than those who survived (n = 7), and a statistically significant positive correlation between HGF and LASV antigen was observed in LF patients (n = 22). Dashed lines and gray shading indicate limits of detection. ALT, alanine aminotransferase; AST, aspartate aminotransferase; D, died; HGF, hepatocyte growth factor; LF, Lassa fever; LASV-Ag, Lassa fever virus antigen; S, survived.

Elevated plasma HGF has correlated with severe coagulopathy, including DIC (*19*). During acute LF, we noted that HGF correlated with markers of liver damage, including total bilirubin (*r_s_* = 0.7510; p<0.0001), alkaline phosphatase (*r_s_* = 0.6499; p = 0.0003), aspartate transaminase (*r_s_* = 0.8383; p<0.0001), and alanine transaminase (*r_s_* = 0.8163; p<0.0001) (Figure 4, panel A). However, HGF alone was a better marker of fatal LF; the mean HGF in survivors was 3.27 ng/mL compared with 23.73 ng/mL in patients who died (p = 0.0005) (Figure 4, panel B). We also found that levels of HGF positively correlated with levels of LASV-Ag (*r_s_* = 0.4554; p = 0.0332).

### Endothelial Activation

**Figure 5:**
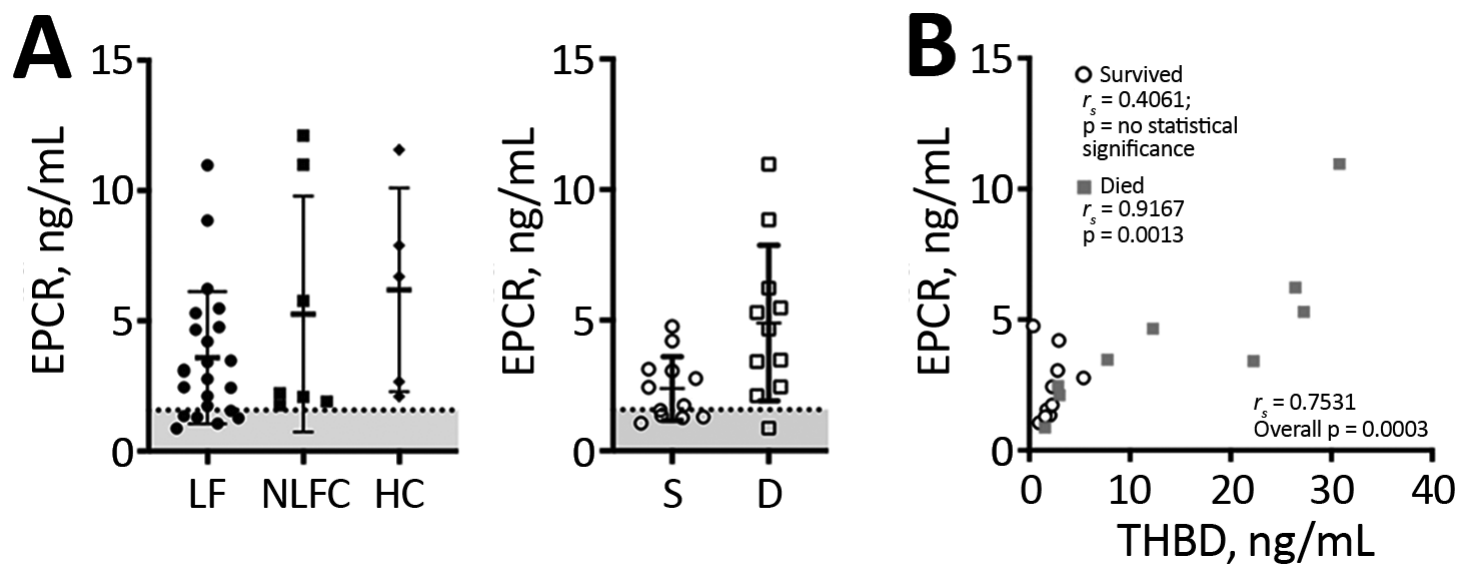
Soluble endothelial protein C receptor plasma levels in patients with acute LF, NLFCs, and HCs, Sierra Leone, 2015–2018. A) EPCR is not statistically significantly different across groups (Kruskal-Wallis p = 0.0889). Error bars show SDs; horizontal lines indicate means. B) EPCR plasma levels correlated with soluble thrombomodulin in patients with acute LF. Patients with higher levels of soluble THBD tended to have higher levels of EPCR (n = 19). When analyzed by survival, a statistically significant positive correlation was found only in fatal LF cases (n = 9). Dashed lines and gray shading indicate limits of detection. D, died; EPCR, endothelial protein C receptor; HC, healthy control; LF, Lassa fever; NLFC, non-LF fever febrile control; THBD, thrombomodulin.

EPCR promotes generation of APC by the thrombin–thrombomodulin complex by recruiting protein C to the endothelial surface (*20*). APC, when associated with the EPCR on endothelial cells, signals through protease-activated receptor 1 (PAR-1) to stabilize endothelial barrier function through an increase in tight junctions, antiapoptotic signals, and suppression of inflammatory cytokines (*21*–*23*). We measured soluble EPCR (sEPCR) to assess whether this pathway is altered during LF. We found no statistically significant differences in sEPCR levels between analyzed groups (Figure 4, panel A), but we observed a statistically significant positive correlation between plasma concentrations of sTHBD and sEPCR (*r_s_* = 0.7351; p = 0.0003). When analyzed by outcome, we only observed this correlation in fatal LF cases (*r_s_* = 0.9167; p = 0.0013; Figure 5, panel B).

**Figure 6:**
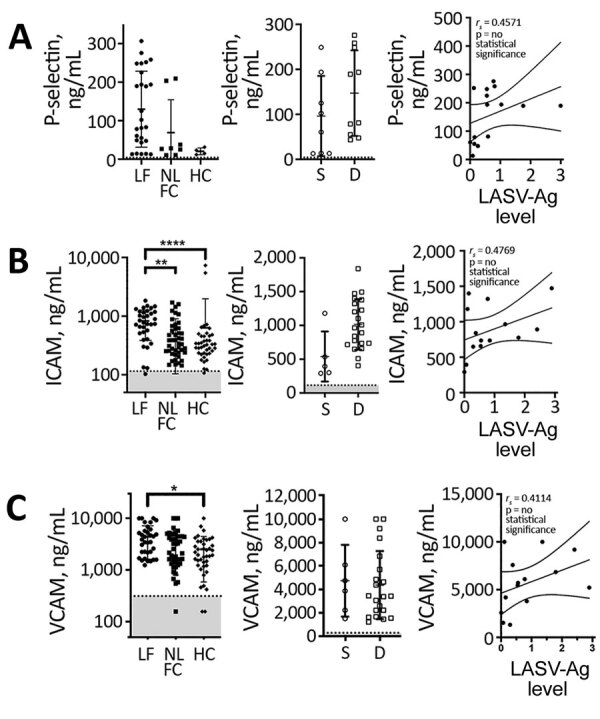
P-selectin and adhesion molecule levels in patients with acute LF, NLFCs, and healthy controls (HCs), Sierra Leone, 2015–2018. A) Differences in soluble P-selectin (CD62P) were statistically significant (Kruskal-Wallis p = 0.0358), but we found no statistically significant differences when comparing groups to each other using Dunn’s multiple comparisons test (left, middle); no statistically significant correlation was observed between P-selectin and LASV-Ag levels (n = 15). B) Statistically significant differences in soluble ICAM levels were noted across all groups (Kruskal-Wallis p<0.0001). ICAM was statistically significantly elevated (****p<0.0001) in acute LF (n = 34) compared with HCs (n = 41) and NLFCs (n = 44; **p = 0.0036). No statistically significant correlation was found between ICAM and LASV antigen (n = 14) C) Statistically significant differences in soluble VCAM levels were observed across all groups (Kruskal-Wallis p = 0.0052). VCAM was statistically significantly elevated (*p = 0.0127) in acute LF (n = 34) compared with HCs (n = 41). No statistically significant differences were observed in acute LF patients who survived (n = 6) compared with those who died (n = 21) and no statistically significant correlation was found between VCAM and LASV-Ag (n = 14). Limits of detection are indicated by dashed lines and gray shading below. Error bars show SDs; horizontal lines indicate means. D, died; HC, healthy controls; ICAM, intercellular adhesion molecule; LF, Lassa fever; LASV, Lassa fever virus; LASV-Ag, Lassa fever virus antigen; NLFC, non–LF febrile controls; S, survived; VCAM, vascular cell adhesion molecule.

Damage to the endothelial barrier causes exposure and secretion of platelet agonists, including collagen and vWF. P-selectin is found on the plasma membrane of platelets after activation, but an alternatively spliced P-selectin isoform lacking the transmembrane domain also is released from endothelial cells and platelet α granules upon activation (*24*,*25*). Plasma levels of soluble P-selectin are elevated during DIC, systemic thrombosis, and platelet consumption (*26*–*29*). We found higher levels of soluble P-selectin (mean 129.7 ng/mL) in LF patients than HCs (mean 20.34 ng/mL), but not statistically significant differences (p<0.05), nor did we find a statistically significant correlation between levels of P-selectin and LASV-Ag (Figure 6, panel A).

Activated endothelia release intracellular adhesion molecule 1 (ICAM-1) and vascular cell adhesion molecule 1 (VCAM-1) (*30*,*31*). We measured soluble ICAM-1 (sICAM-1) and soluble VCAM-1 (sVCAM-1) and found LF patients had statistically significantly higher levels of both markers (mean sICAM-1 829.1 ng/mL; mean sVCAM-1 4,388 ng/mL) compared with HCs (mean sICAM-1 633.5 ng/mL, p<0.0001; mean sVCAM-1 2,517 ng/mL, p = 0.0127) (Figure 6, panels B, C). We also observed statistically significant differences in sICAM-1 levels between HCs and fatal LF cases (mean sICAM-1 1,015 ng/mL; p = 0.0001) (Figure 4, panel B), but not with sVCAM-1 (Figure 6, panel C). We found no statistically significant correlation between sICAM-1 or sVCAM-1 and LASV-Ag (Figure 6, panels B, C).

### Platelet Dysfunction 

An inhibitor of platelet aggregation has been observed in the plasma of patients with severe LF and Argentine hemorrhagic fever, which is caused by the arenavirus Junin (*7*,*32*,*33*). The inhibition is platelet extrinsic and aggregation inhibition can be observed in plasma–platelet mixing experiments by using either autologous platelets from the acute case or platelets from healthy persons. We observed the same phenomenon in a mouse model of lethal arenavirus infection (*37*). We performed platelet aggregometry on a 1:1 mix of platelet-rich plasma from a healthy person collected in citrated tubes and dialyzed plasma from LF patients. EDTA inhibits platelet aggregation. KGH biorepository samples were collected in EDTA. Therefore, we dialyzed plasma used in aggregation assays to remove EDTA. Our unpublished observations in mice indicate EDTA can be dialyzed out of the plasma while retaining the inhibitory function of the platelet aggregation inhibitor.

**Figure 7:**
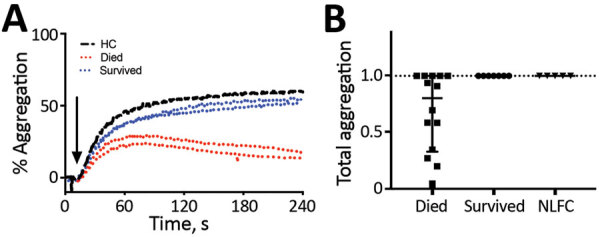
Representative platelet aggregometry performed on a 1:1 mix of platelet-rich plasma from a healthy control (HC) participant and platelet-poor plasma dialyzed to remove EDTA from either healthy controls or acute Lassa fever (LF) patients, Sierra Leone, 2015–2018. Aggregation was stimulated by addition of 5 μmol ADP. Plasma from fatal LF cases caused a decrease in aggregation at 4 min compared with peak aggregation, but plasma from LF survivor and non–LF febrile controls (NLFCs) showed no disaggregation by 4 min. A) Percent aggregation over 4 min. B) Aggregation at 4 min divided by the maximum aggregation in assays by using plasma from 14 fatal LF cases, 7 nonfatal LF cases, and 5 NLFC cases. Only assays using plasma from fatal cases showed disaggregation by the experimental endpoint. Error bars shows SD; horizontal lines indicate means. HC, healthy control; LF, Lassa fever; NLFC, non-LF febrile control.

Plasma samples varied in their turbidity and we could not reliably compare aggregation curves between samples. However, when platelets are agonized by ADP in the presence of the inhibitor, a characteristic disaggregation curve is observed (*32*–*34*). Platelet aggregation and subsequent disaggregation after ADP addition is independent of sample turbidity (Figure 7, panel A). Thus, we assessed the relative difference between peak aggregation and total aggregation 4 min after ADP addition. Platelet disaggregation only occurred in samples from fatal LF cases (Figure 7, panel B).

## Discussion

We identified differences in plasma markers between nonfatal and fatal LF that imply loss of homeostasis, alterations in the protein C pathway, and platelet dysfunction likely contribute to weakened endothelial barriers observed in fatal LF cases. The increased sTHBD we noted in fatal LF is consistent with vascular dysfunction seen in fatal LF cases. The concomitant increase in sEPCR suggests endothelial expression of both receptors of the protein C pathway were affected, pointing toward a potential impaired ability of the endothelium to generate APC and mediate APC cellular function, which includes stabilizing endothelial barrier function.

APC and EPCR also can inhibit NLRP3 inflammasome and apoptosis, which could contribute to Lassa-induced immunopathology. Studies show elevated interleukin 1 beta during later stages of LASV infection in cynomologous macaques (*35*), but this and other markers of inflammasome activation have not been well characterized during human infection. Recombinant APC (rAPC) therapy has had mixed results. After initial positive preclinical results (*36*), rAPC was ineffective in treating sepsis (*37*). However, rAPC given to Zaire ebolavirus–infected primates delayed death by several days (*38*). Because we could not measure expression of THBD and EPCR on the surface of endothelial cells, we do not know whether the signaling capacity of the APC-EPCR-PAR-1 pathway changes during LF.

Other mechanisms for disruption of endothelial barrier function have been proposed and also might play a role in LF. LASV can alter the biosynthesis of its cellular receptor, α-dystroglycan (*39*) and disrupt endothelial connections to the extracellular matrix by displacing laminin (*40*–*42*). Immune-mediated targeting of infected endothelial cells causes the pulmonary vascular permeability observed in our mouse model of lethal arenavirus infection (*34*,*43*). Cytokine storm has been implicated as a mechanism of capillary leak, but tumor necrosis factor α, a cytokine responsible for increasing vascular permeability, rarely has been observed in plasma of animal arenavirus models (*35*,*44*) or human LF cases (*45*–*47*).

**Figure 8:**
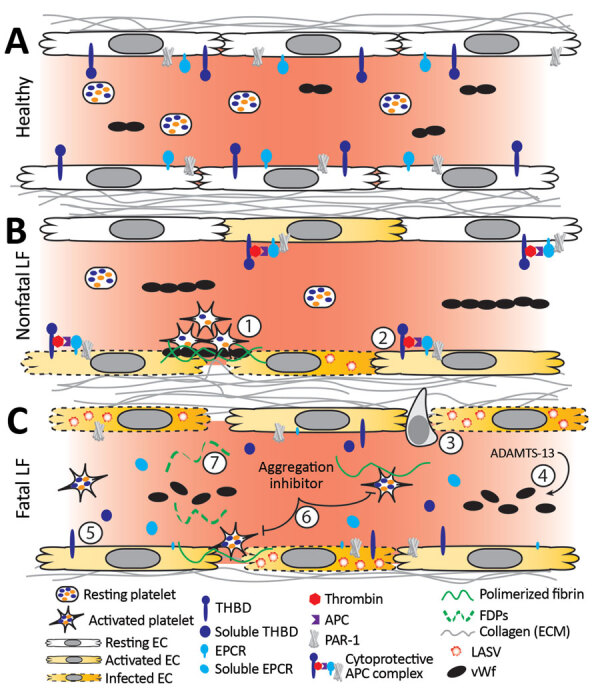
Working model of endothelial barrier function during time-of-admission for Lassa fever showing differences in function between healthy endothelium, and endothelium in nonfatal and fatal Lassa fever, Sierra Leone during 2015–2018. A) Healthy endothelium; resting platelets circulate and lower molecular weight vWF multimers are constitutively secreted by the endothelium. B) Endothelium barrier function in non-fatal Lassa fever. 1) Immune mediated damage near infected endothelial cells leads to collagen exposure, fibrin deposition, platelet activation, endothelial activation, and release of ultra-high molecular weight vWF from endothelial cells and platelets. Functional coagulation and platelet responses lead to endothelial repair. 2) Endothelial surface bound thrombomodulin and EPCR lead to activation of protein C; PAR-1 activation strengthens endothelial tight junctions, increases endothelial survival, and dampens the inflammatory response. C) Endothelium barrier function in fatal Lassa fever. 3) Increased endothelial infection has been observed in fatal LF cases. Increased immune infiltrates likely stress the endothelial barrier and lead to increased endothelial activation, evidenced by increased soluble ICAM, P-selectin, and EPCR. 4) Ultra-high molecular weight vWF multimers are broken down by ADAMTS-13, likely leading to the increased vWF ELISA signal observed during fatal LF. 5) Increases in soluble thrombomodulin and EPCR likely leave less surface bound forms, inhibiting the ability of endothelial cells to activate cytoprotective pathways through PAR-1 and increasing their susceptibility to immune mediated destruction. 6) Our in vitro aggregation studies show that platelets in the presence of the aggregation inhibitor become activated and change shape in response to platelet agonists but fail to maintain the aggregated state. These data are consistent with the inhibition of the release of α or dense granules or their contents, thereby inhibiting certain aspects of primary hemostasis, clot formation, and endothelial repair. 7) The state of fibrinolysis during fatal LF is unclear. Increased PAI-1 suggests an inhibition of fibrinolysis, but D-dimers and other FDPs are only formed during plasmin mediated breakdown of fibrin. Likely, increased platelet activation leads to an increase of total fibrin formation. Platelet dysfunction can lead to weaker hemostatic plugs which then leave fibrin more susceptible to cleavage by plasmin. APC, activated protein C; EC, endothelial cell; EPCR, endothelial protein C receptor; FDP, fibrin degradation product; ICAM, intercellular adhesion molecule; LASV, Lassa fever virus; PAI-1, plasminogen activator inhibitor 1; PAR-1, protease-activated receptor 1; plasminogen activator inhibitor 1; THBD, thrombomodulin; vWF, von Willebrand factor.

Diseases that manifest as systemic endothelial dysfunction, such as LF, lead to disruption in homeostasis through exposure of TF, triggering coagulation cascade and platelet activation on the basal side of the endothelium. Observation of a platelet aggregation inhibitor only in fatal cases implies this factor is a potential contributor to the bleeding and vascular permeability characteristic of severe, fatal disease. In response to ADP and collagen, platelets exposed to the aggregation inhibitor begin to aggregate normally (*32*; Figure 8, panel A), indicating platelet activation is maintained. However, the initial aggregation is followed by disaggregation, suggesting an inhibition of platelet degranulation. Release of platelet granule contents is necessary for the second wave of platelet activation that sustains aggregation. The contents of these granules also are vital for coagulation, angiogenesis, wound repair, and inflammation; thus, inhibition of their release can have systemic effects.

Prior studies concluded coagulation dysfunction and DIC are not features of acute LF (*48*,*49*). We observed D-dimers and tPA trending higher, but these indicators of changes in fibrinolysis were not statistically significant. Moreover, we could not determine whether patients had DIC by the standardized diagnostic scoring system (*50*). Nevertheless, a positive correlation between increased D-dimers and LASV-Ag without an increase in TAT complexes suggest an atypical coagulation dysfunction. However, our data support pathologic activation of coagulation indicated by increased circulating TF, sTHBD, and vWF. Presence of a platelet aggregation inhibitor might complicate coagulation dysfunction during LF further by disrupting or weakening active thrombus formation, leading to changes in blood coagulation parameters that appear similar to DIC but without thrombus formation in the microvasculature. Biochemical identification of the inhibitor and imaging studies to identify microthrombi in the vasculature of animals and humans could help clarify whether, and to what extent, DIC occurs during severe LF.

Our studies provide a comprehensive analysis of coagulation and endotheliopathy in human LF cases. These data only offer a snapshot of hemostatic changes; we cannot rule out resolution of dysregulated hemostasis in nonfatal LF patients before admission. Certain clinical and epidemiologic information, such as date of symptom onset and outcomes for patients not admitted to the Lassa ward, was not available. Moreover, unavailability of samples and ELISA kits in Sierra Leone limited more extensive analyses of our data, specifically in cases in which analytes trended but did not reach statistical significance. More robust patient data and sample collection could help define hematological dysfunction during disease progression and correlate these markers with signs, such as facial and pulmonary edema.

In conclusion, we propose a model in which immunopathology causes disruption to the endothelial barrier (Figure 8). The combination of endothelial damage and an inability to repair it could contribute to the vascular permeability observed in most severe LF cases. In addition, the cytoprotective APC pathway and platelet aggregation, mechanisms that normally protect and maintain endothelial cell integrity, are disrupted during LASV infection. Further studies incorporating serial samples and samples collected earlier in the disease course are needed in animal models and humans to elucidate the molecular mechanisms underlying vascular dysfunction during severe LF. However, our data forms a basis for investigation into specific pathways that could provide targets of intervention to minimize the effects of host immunopathology and enable the immune system adequate time to clear the virus.
